# Supplementary material for: Unraveling the architecture of major histocompatibility complex class II haplotypes in rhesus macaques
Source: Genome Res. 2024 Nov;34(11):1811–24. doi: 10.1101/gr.278968.124 (PMC11610599; doi:10.1101/gr.278968.124)
Supplement: Supplement 1 [file Supplemental_Material.zip › Supplemental_Fig_S2.pdf]

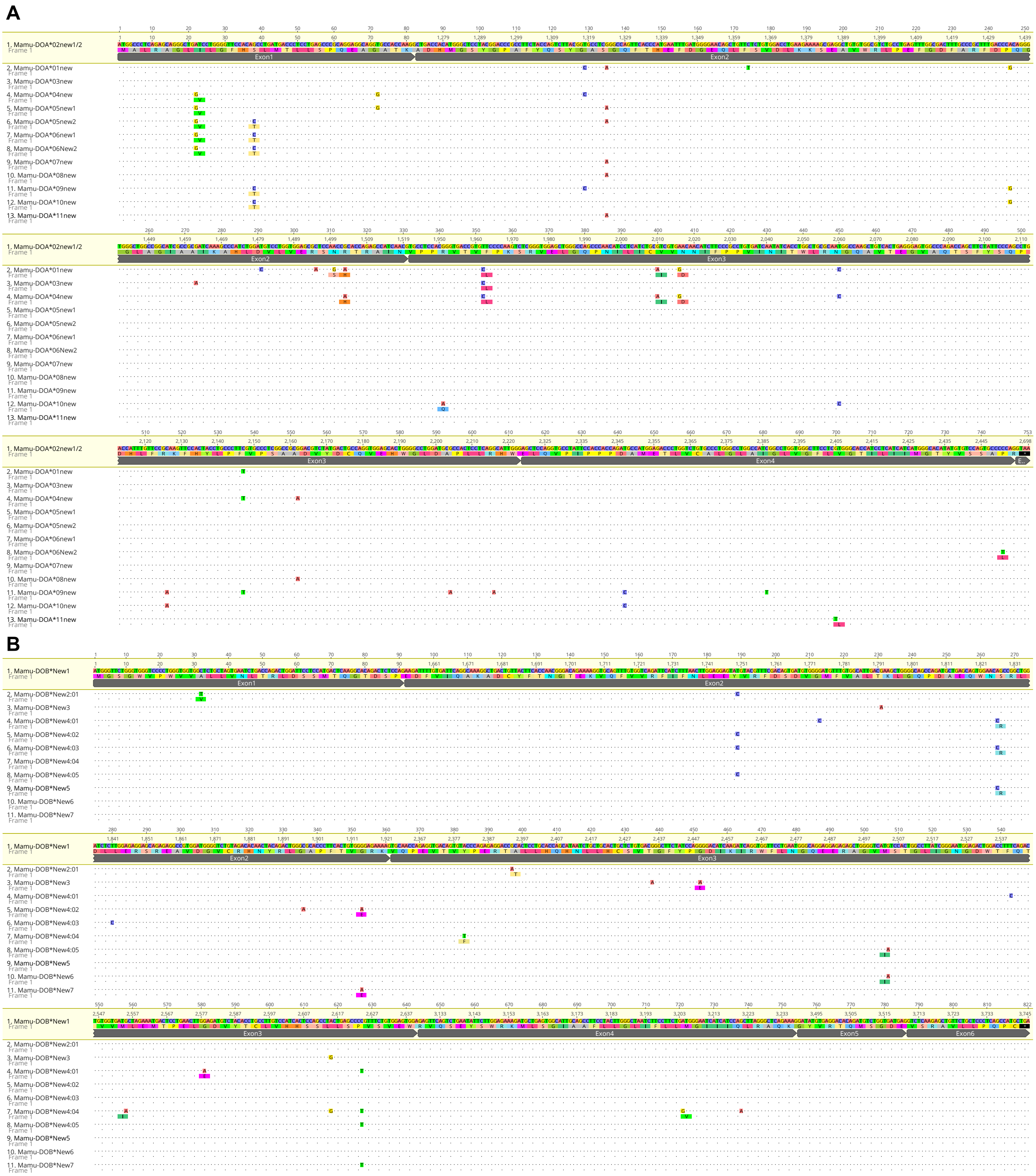

**Supplemental Figure 2. Sequence alignments of *Mamu-DOA* and *-DOB* exons.** One *Mamu-DOA* (a) and one *-DOB* (b) sequence were used as references, to which all other identified sequences were aligned using MUSCLE. All differences in comparison to the reference sequence are indicated at the nucleotide and amino acid level, whereas similarities are indicated by dots.
