# Supplementary material for: Unraveling the architecture of major histocompatibility complex class II haplotypes in rhesus macaques
Source: Genome Res. 2024 Nov;34(11):1811–24. doi: 10.1101/gr.278968.124 (PMC11610599; doi:10.1101/gr.278968.124)
Supplement: Supplement 1 [file Supplemental_Material.zip › Supplemental_Fig_S3.pdf]

A

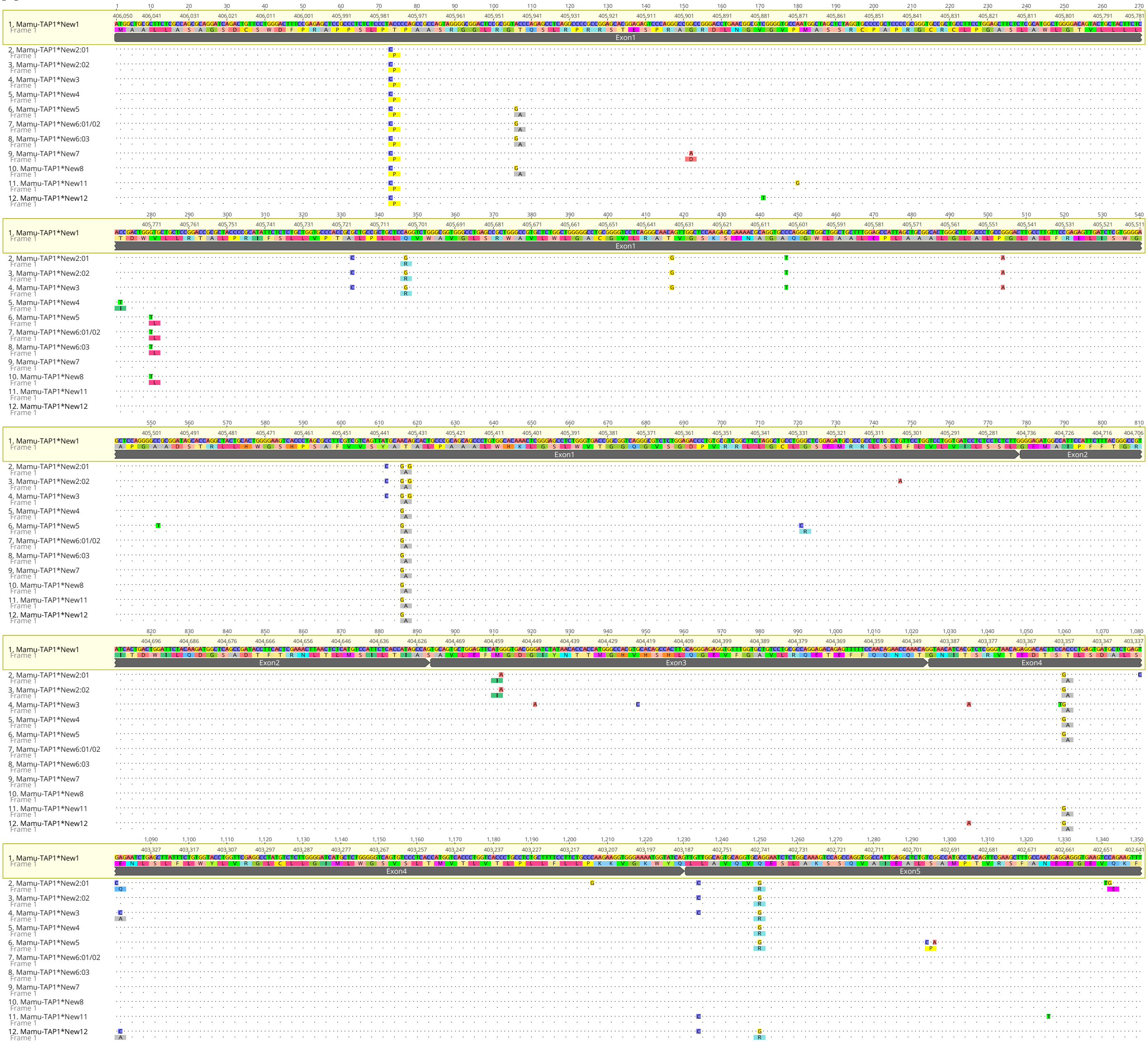

Supplemental Figure 3. Sequence alignments of *Mamu-TAP1* and *-TAP2* exons. One *Mamu-TAP1* (a) and one *-TAP2* (b) sequence were used as references, to which all other identified sequences were aligned using MUSCLE. All differences in comparison to the reference sequence are indicated at the nucleotide and amino acid level, whereas similarities are indicated by dots. This figure continues on the next pages.

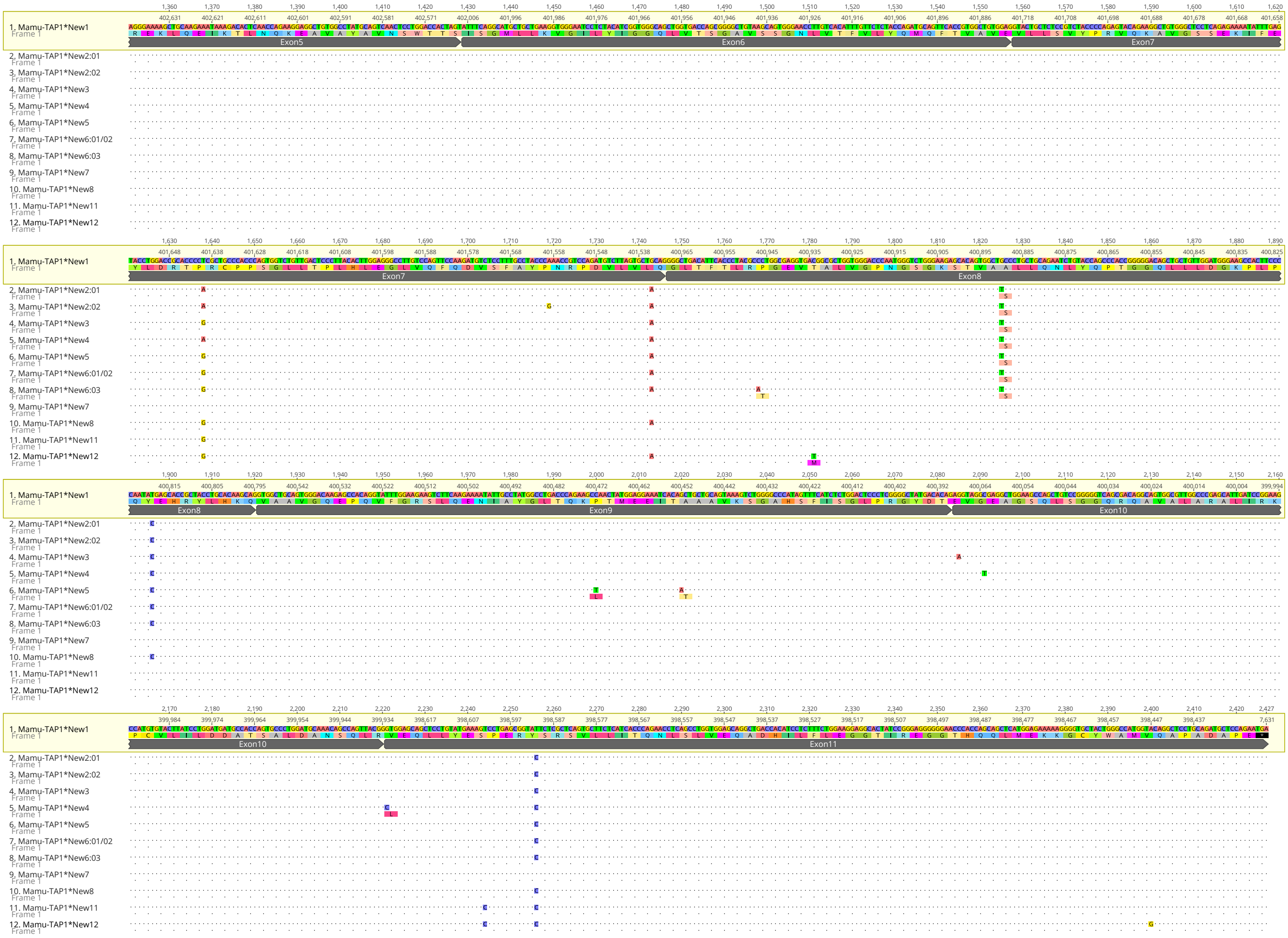
1, Mamu-TAP1*New1
2, Mamu-TAP1*New2:01
3, Mamu-TAP1*New2:02
4, Mamu-TAP1*New3
5, Mamu-TAP1*New4
6, Mamu-TAP1*New5
7, Mamu-TAP1*New6:01/02
8, Mamu-TAP1*New6:03
9, Mamu-TAP1*New7
10, Mamu-TAP1*New8
11, Mamu-TAP1*New11
12, Mamu-TAP1*New12
Frame 1
Exon5
Exon6
Exon7
Exon8
Exon9
Exon10
Exon11

B

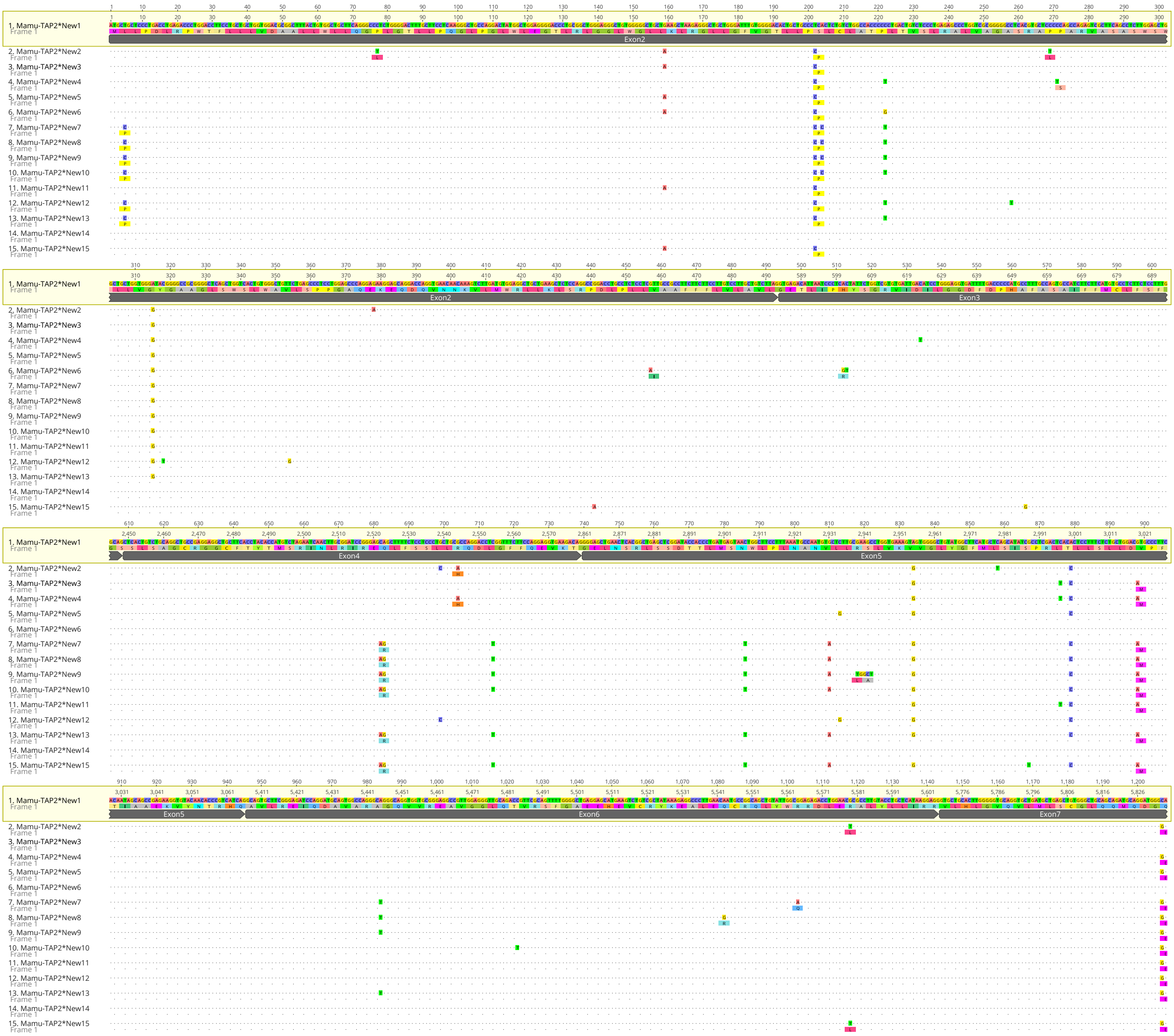
1. Mamu-TAP2*New1
2. Mamu-TAP2*New2
3. Mamu-TAP2*New3
4. Mamu-TAP2*New4
5. Mamu-TAP2*New5
6. Mamu-TAP2*New6
7. Mamu-TAP2*New7
8. Mamu-TAP2*New8
9. Mamu-TAP2*New9
10. Mamu-TAP2*New10
11. Mamu-TAP2*New11
12. Mamu-TAP2*New12
13. Mamu-TAP2*New13
14. Mamu-TAP2*New14
15. Mamu-TAP2*New15
Frame 1
Exon2
Exon3
Exon4
Exon5
Exon6
Exon7

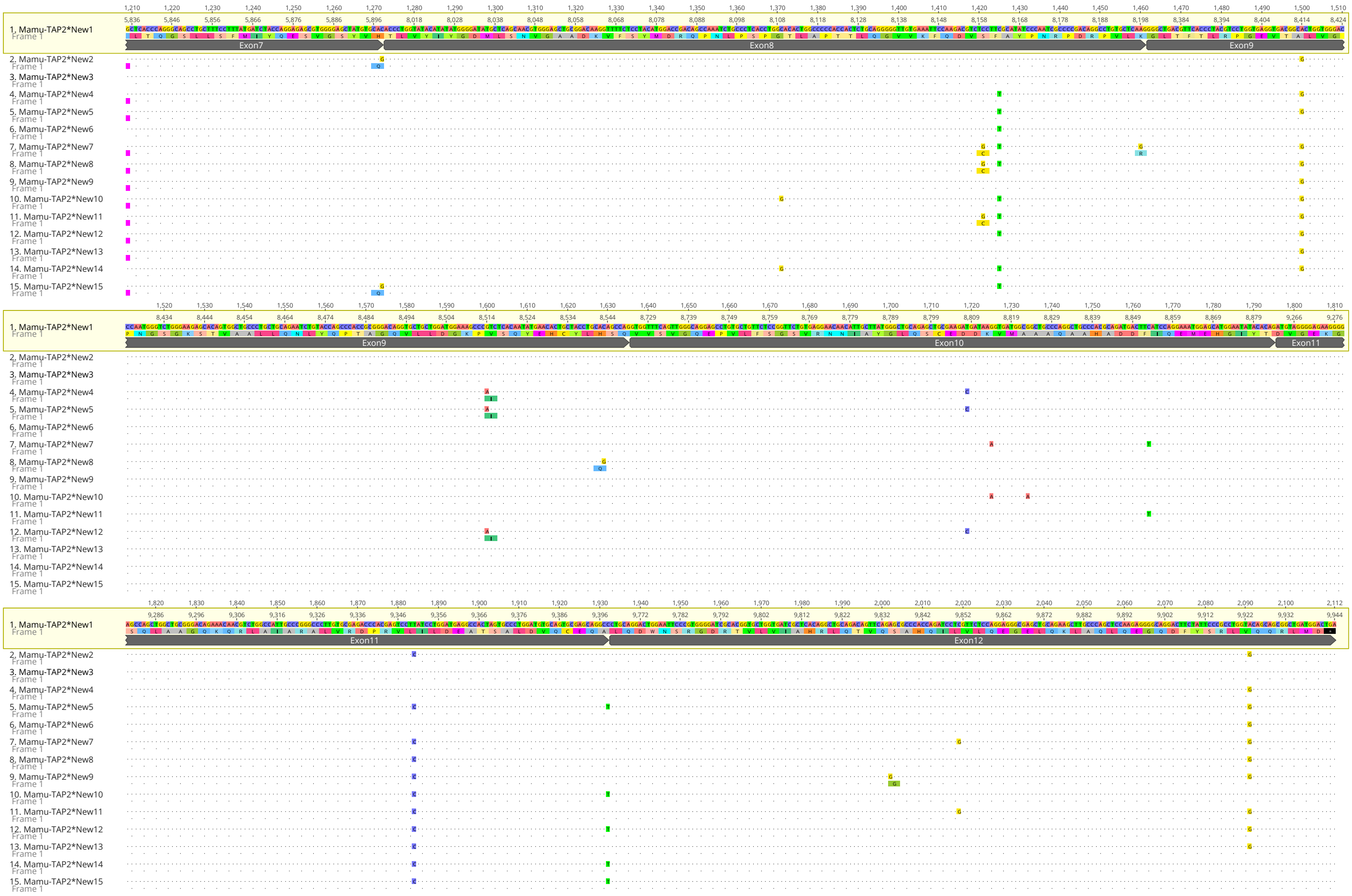

1. Mamu-TAP2*New1
2. Mamu-TAP2*New2
3. Mamu-TAP2*New3
4. Mamu-TAP2*New4
5. Mamu-TAP2*New5
6. Mamu-TAP2*New6
7. Mamu-TAP2*New7
8. Mamu-TAP2*New8
9. Mamu-TAP2*New9
10. Mamu-TAP2*New10
11. Mamu-TAP2*New11
12. Mamu-TAP2*New12
13. Mamu-TAP2*New13
14. Mamu-TAP2*New14
15. Mamu-TAP2*New15
Frame 1
Exon7
Exon8
Exon9
Exon10
Exon11
Exon12
