# Supplementary material for: Unraveling the architecture of major histocompatibility complex class II haplotypes in rhesus macaques
Source: Genome Res. 2024 Nov;34(11):1811–24. doi: 10.1101/gr.278968.124 (PMC11610599; doi:10.1101/gr.278968.124)
Supplement: Supplement 1 [file Supplemental_Material.zip › Supplemental_Fig_S4.pdf]

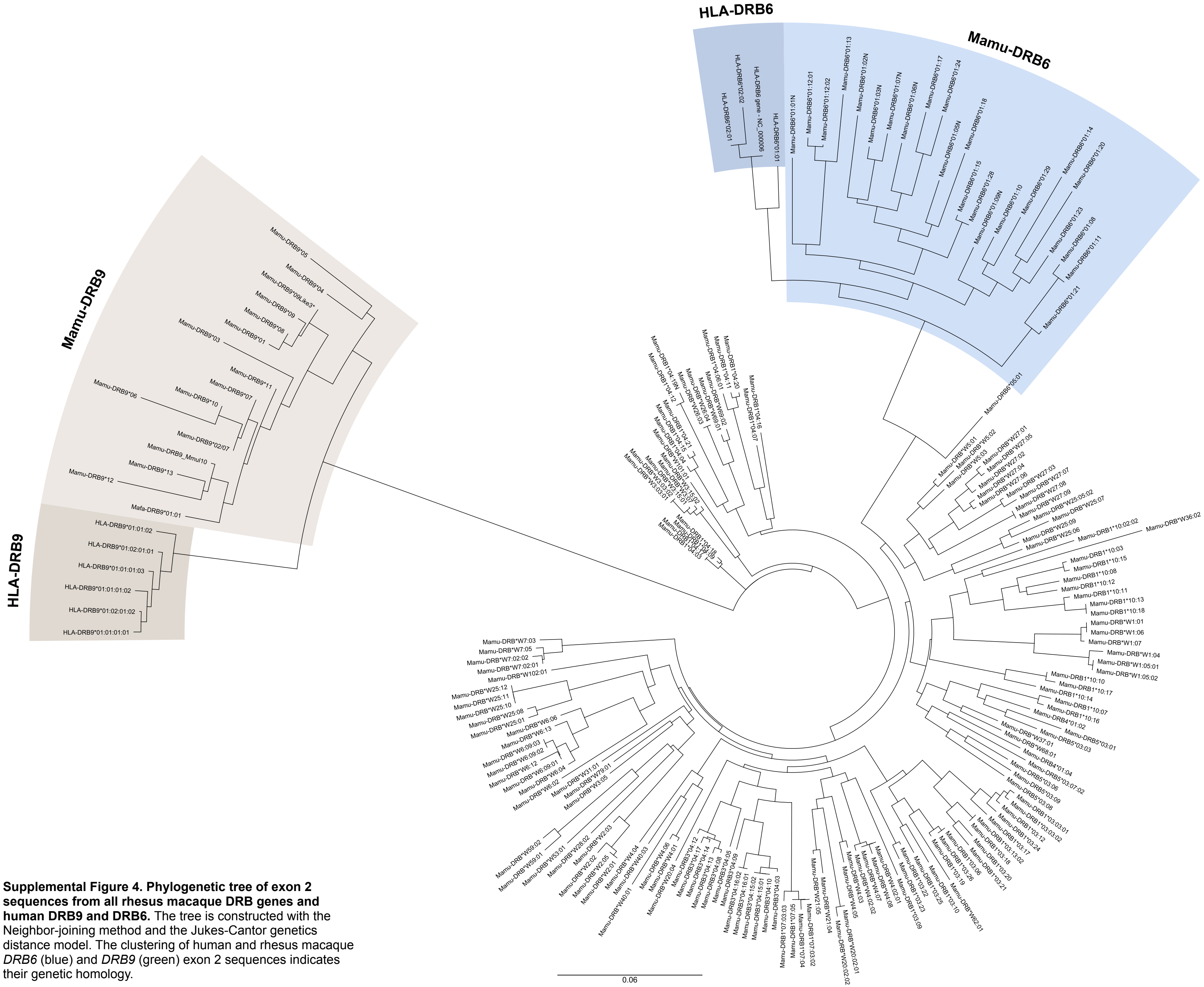

Supplemental Figure 4. Phylogenetic tree of exon 2 sequences from all rhesus macaque DRB genes and human DRB9 and DRB6. The tree is constructed with the Neighbor-joining method and the Jukes-Cantor genetics distance model. The clustering of human and rhesus macaque *DRB6* (blue) and *DRB9* (green) exon 2 sequences indicates their genetic homology.
