# Supplementary material for: Unraveling the architecture of major histocompatibility complex class II haplotypes in rhesus macaques
Source: Genome Res. 2024 Nov;34(11):1811–24. doi: 10.1101/gr.278968.124 (PMC11610599; doi:10.1101/gr.278968.124)
Supplement: Supplement 1 [file Supplemental_Material.zip › Supplemental_Fig_S5.pdf]

A

B

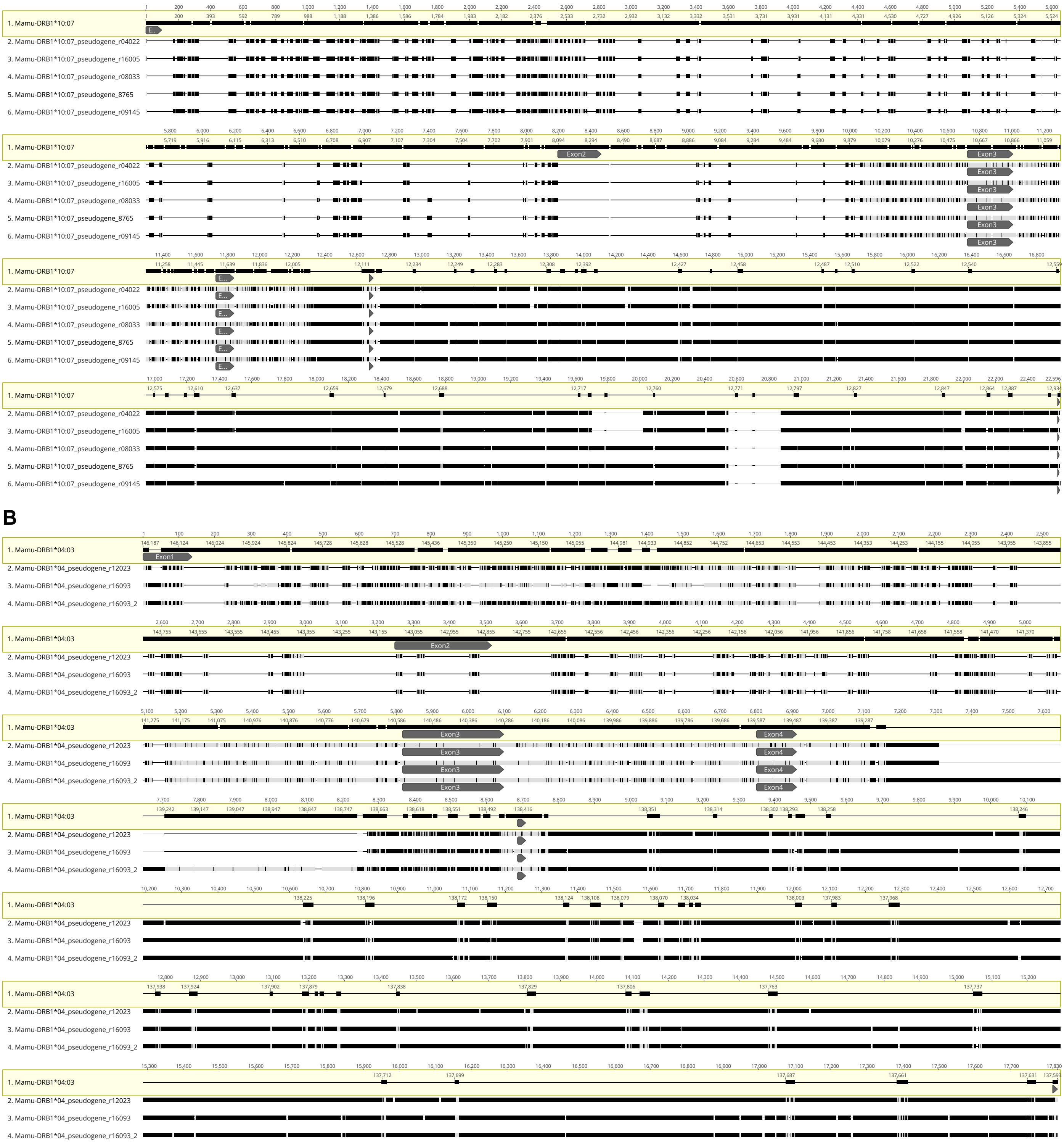

Supplemental Figure S5. Alignment of *DRB* pseudogene sequences to the most similar functional *DRB* gene. A genomic nucleotide sequence of a functional *Mamu-DRB1*10:07* (A) and -DRB1*04 (B) are used as reference sequence, to which multiple sequences of related pseudogenes are aligned using MAFFT. Similarities to the reference sequence are highlighted in grey, whereas deviations are indicated in black. A black line reflects gaps in the alignment. The exons are represented as grey arrows. The pseudogenes display similarity to the functional counterpart only from the end of intron 2 to slightly beyond exon 5.
