# Supplementary material for: Unraveling the architecture of major histocompatibility complex class II haplotypes in rhesus macaques
Source: Genome Res. 2024 Nov;34(11):1811–24. doi: 10.1101/gr.278968.124 (PMC11610599; doi:10.1101/gr.278968.124)
Supplement: Supplement 1 [file Supplemental_Material.zip › Supplemental_Table_S1.pdf]

Supplemental Table 1. Overview of all *MHC* class II haplotypes identified in the rhesus macaque panel studied. The haplotypes are numbered according to their *DR* region configurations, which were previously designated [25]. The two haplotypes that are marked with an asterisk (*) were previously designated as 12 and 13a by Doxiadis *et al.* (2013), but they are found to share an identical region configuration according to our new assemblies. Two haplotypes reflect novel region configurations and are designated as "New". Two haplotypes were resolved from animals that have a mixed origin (e.g. Indian-Chinese, Indian-Burmese). The *DR* region, comprising the *DRA* locus and the diverse *DRB* gene combinations, is shaded in yellow. Known pseudogenes in the *DR* region are indicated by a grey text color. The *DQ* and *DP* genes are indicated with a blue and an orange background, respectively. Novel alleles are indicated with a "new" designation. Some parts of the *MHC* class II haplotypes could not be resolved due to low coverage, and are marked as Not determined (N.d.). Four genes were determined at low coverage (< 3X) and are marked by the blue text color. A *DP* tandem duplication was found in animal r16005, whereas all other *MHC* class II haplotypes only vary in gene content in their *DRB* region.

#	Animal	DRA	DRB9	DRB	DRB	DRB	DRB	DRB	DRB	DRB	DQA	DQB	DOB	TAP2	PSMB8	TAP1	PSMB9	DMB	DMA	BRD2	DOA	DPA	DPB	DPA	DPB	DPB2
1a	r14109, EAW	01:02:03:01	05	DRB1*04:06:01:02	DRB5*03:01:01						26:02:01	27:02	new1:01	new14	new6	new1:01	new1	03:01New2	02:02:01new1	new1:01	02new1	02:02:01	07:01			01new7
2	r08033	01:04:02:01	03new	DRB3*04:03	DRB*W003:05:01						24:01:01	18:10	new4:02	N.d.	N.d.	new6:03	new5	03:02new4	02:01:04new	new1:03:04	06new	02:05:01	15:01			01new2
3a	r06048	01:05:01:01	13	DRB1*03:03	DRB6*01:03N	DRB1*10:07					26:01	18:01	new1:01	new3	new5	new6:01	new5:02	03:01new2	02:02:01new2	New1:01	02new1	02:02:01	07:01			01:03
3b	r16093	01:05:01:01	13	DRB1*03:12	DRB6*01:03N	DRB1*10:07					26:01	18:01	new4:04	new1	new7	new1:01	new1	03:01new1	02:02:01new2	new1:01	02new1	02:02:01	07:01			01new11
3d	9066	01:02:01new	06	DRB1*03:18	DRB6*01:07N	DRB1*10:03					01:02	06:05	new1:03	new11	new12	new6:03	new5	03:02new4	02:01:04new	new1:03:03	06new2	02:05:01	15:01			01new12
3e	r07112, r06048,r09145	01:04:01new	01	DRB1*03:06	DRB6*01:03N	DRB1*10:07					26:01	18:01	new1:01	new3	new5	new6:01	new5:02	03:02new2	02:02:02new	new1:02:01	08new	02:08	06:04			01new5
4	r16005	01:02:04:01	01	DRB1*03:09	DRB6*01:01N	DRB*W002:01					01:04:01	06:01	new5	new5	new8	new3	new3	03:02new4	02:01:04new	new1:03:01	05new1	11:01	16:01	07new	08new	01new9
4	r04022	01:02:04:01	01	DRB1*03:09	DRB6*01:01N	DRB*W002:01					01:04:01	06:01	new5	new12	new10	new3	new3	03:02new	02:01:04new3	new1:02:02	03new	02:04	08:01			01new1:02
4	r02034, EAW	01:02:04:01	01	DRB1*03:09	DRB6*01:01N	DRB*W002:01					01:04:01	06:01	new5	new12	new10	new7	new2	03:02new	new2	new1:03:02	05new2	02:05:01	15:01			01new12
5	RiN14	01:02:02new	07	DRB1*04:03	DRB6*01:07N	DRB*W005:01					24:02	28:01:01	new4:05	new9	new2	new5	new5	03:01new1	02:02new	new3	01new	02:03	08:02			01new5
5	r04022,8765	01:02:02new	07	DRB1*04:03	DRB6*01:07N	DRB*W005:01					24:02	28:01:01	new6	new13	new2	new8	new5	03:02new2	02:01:04new4	new5	10new	06:01:01	01:01			01new6:02
6	9066	01:02:01:03	11	DRB*W003:03	DRB6*01:14	DRB*W004:01					23:01	18:02	new1:02	new10	new1	new2:02	new4	03:02new7	02:02new	new2:01	04new	04:03:01	02:01			01new3
8	r11021	01:03:01:01	12	DRB6*01:28	DRB*W028:01	DRB5*03:07:02					24:04	15:03	new2:01	new4	new7	new4	new8	03:02:01ext	02:01:01ext	new1:01	02new2	04:01	03:02			01new4
11	r11021	01:03:01new	09new	DRB*W020:02:01	DRB6*01:12:02N	DRB*W025:01					26:02:02	27:02	new4:04	new4	new7	new3	new3	03:02new	02:01:04new	new1:02:01	03new	02:04	08:01			01new1
New18	r02034	01:04:01new	08	DRB*W020:02:01	DRB6*01:02N	DRB*W007:02:01					26:02:02	27:02	new4:04	new4	new7	new3	new3	03:02new	02:01:04new5	new1:03:03	06new	02:08	06:04			01new5
10	r12023	01:02:07:01	10	DRB3*04:05	DRB6*01:23	DRB1*07:01	DRB5*03:03				26:03	15:01:01	new1:01:02	new7	new1	new2	new9	03:02:01ext	02:01:01ext	new1:01	02new2	02:07:01	05:01			01new10
9	r16093	01:02:05:01	09	DRB1*04:04:01	DRB6*01:20	DRB*W003:07	DRB6*01:02N	DRB*W007:02			26:02:02	27:02	new1:01	new4	new6	new3	new3	03:02new2	02:02new2	new1:03:02	05new2	02:05:01	15:01			01new12
12*	RiN14	01:02:01new	06	DRB1*03:18	DRB6*01:05N	DRB*W006:04	DRB6*01:18	DRB*W006:03			24:05	15:02	new4:03	new8	new1	new2:02	new4	03:02new7	02:01:04new2	new2:01	04new	04:03:01	02:01			01new13
12*	r12023	01:02:01new	06	DRB1*03:18	DRB6*01:05N	DRB*W006:04	DRB6*01:18	DRB*W006:03			24:05	15:02	new4:03	new8	new1	new2:02	new4	03:02new	02:01:04new	new1:01	05new2	02:05:01	15:01			01new2
14	r09145, r14109	01:04new	03	DRB3*04:10	DRB6*01:07N	DRB*W004:02:01	DRB6*01:16N	DRB*W027:01			01:09	06:09	new3	new6	new3	new11	new7	03new5	new1	new4	09new	07:10	19:06			01new8
15	r08033	01:03:01:01	04	DRB*W021:04	DRB6*01:11N	DRB*W006:06	DRB6*01:22	DRB*W026:03			01:05:01	06:02:01	new4:01	new2	new4	new6:02	new5	03:02new2	02:02:02new	new1:02:01	08new	02:08	06:04			01new5
16	96009, r07112	01:02:06:01	02	DRB1*03:10	DRB6*01:06N	DRB*W001:01	DRB6*01:17	DRB*W006:09	DRB6*01:18	DRB*W006:02	23:02	18:04	new1:01	new1	new9	new1:02	new6	03:02new	02:01:04new	new1:02:01	07new	06:01:01	01:01			01new6
New17	96009 (Burmese)	01:02new	02	DRB4*01:02	DRB5*03:06						01:07	06:10	new1:01	new12	new11	new9	N.d.	03:02new2	02:02new	N.d.	04new	02:08	06:04			01new5
2	8765 (Chinese)	01:02:03new	03	DRB3*04:11	DRB*W003:05:01						26:10	15:01:02	New7	New15	New13	New12	new10	03new6	new3	new6	11new	04:02	02:02			01new14
