# Supplementary material for: Unraveling the architecture of major histocompatibility complex class II haplotypes in rhesus macaques
Source: Genome Res. 2024 Nov;34(11):1811–24. doi: 10.1101/gr.278968.124 (PMC11610599; doi:10.1101/gr.278968.124)
Supplement: Supplement 1 [file Supplemental_Material.zip › Supplemental_Table_S2.pdf]

Supplemental Table S2. Raw and analyzed data of the rhesus macaque *MHC* class II haplotypes. The ENA accession numbers are listed for the raw ONT read files and the assembled and annotated haplotype files per animal. The *MHC-DRB* haplotype numbers correspond to the haplotypes illustrated in Figure 5 and Supplemental Table S1.

Animal	Raw ONT read files accession number	*MHC-DRB* haplotype #	Haplotype accession number
8765	ERR12785015	9	OZ023134
		9	OZ023132
9066	ERR12779146	3D	OZ023133
		6	OZ023839
R02034	ERR12778894	4	OZ023130
		18	OZ023727
96009	ERR12778853	9	OZ023106
		16	OZ023126
R04022	ERR12778673	4	OZ023131
		9	OZ023134
EAW	ERR12778399	1A	OZ023127
		4	OZ023130
R06048	ERR12778375	3A	OZ023136
		3E	OZ023137
R07112	ERR12778351	3E	OZ023137
		16	OZ023126
R08033	ERR12778349	2	OZ023128
		15	OZ023936
R09145	ERR12778348	3E	OZ023137
		14	OZ023107
R12023	ERR12778257	10	OZ023728
		12	OZ023104
R11021	ERR12778255	8	OZ023935
		11	OZ023840
R16005	ERR12778253	4	OZ023129
		4	OZ023129
R16093	ERR12778242	3B	OZ023135
		9	OZ023102
R14109	ERR12778241	1A	OZ023127
		14	OZ023107
RiN14	ERR12771506	9	OZ023103
		12	OZ023105
